# The thromboelastogram is confounded by hematocrit in clinical samples tested on both mechanical and acoustic platforms

**DOI:** 10.3389/fmed.2024.1421727

**Published:** 2024-11-13

**Authors:** Aaron S. Hess

**Affiliations:** ^1^Department of Anesthesiology, University of Wisconsin, Madison, WI, United States; ^2^Department of Pathology and Laboratory Medicine, University of Wisconsin, Madison, WI, United States

**Keywords:** hemostasis, thromboelastogram, red blood cells, fibrinogen, hematocrit

## Abstract

Red blood cells are critical participants in normal hemostasis, but *in vitro* experiments have shown that the thromboelastogram (TEG) maximum amplitude has a paradoxical inverse relationship with hematocrit. This study reviewed all samples at a single academic institution where any mechanical (TEG 5000) or acoustic (TEG 6s) TEG was drawn within 5 min of hematocrit measurement. A total of 7,176 samples were identified using complete TEG and conventional coagulation test data (6,384 mechanical, 744 acoustic, and 48 both). In the primary analysis, hematocrit was negatively associated with the maximum amplitude on both mechanical and acoustic platforms after adjusting for relevant confounders. This suggests that the thromboelastogram may misrepresent the contribution of red blood cells in normal hemostasis.

## 1 Introduction

Viscoelastic tests (VETs) are a family of coagulation assays that measure clot shear modulus over time and provide a view of several phases of hemostasis *ex vivo* (1, 2). In typical VETs such as the thromboelastogram (TEG), whole blood is exposed to an activator and then subjected to repeated stress with sound waves or mechanical torsion (3). As the clot evolves, clot initiation, propagation, stabilization, and lysis can be assessed from a single tracing (4). Although they are popular (5), fast (2), and usable at point-of-care, VETs such as the TEG do not offer a complete or reproducible picture of coagulation (6). Many acquired and inborn defects, including Von Willebrand diseases, platelet function disorders, antiplatelet therapies, and antithrombin activity are not assessed by VETs (7, 8). VETs also appear to be insensitive to the hemostatic effects of erythrocytosis and anemia (9).

Anemia is associated with an increased risk of bleeding (10–12). Red blood cells (RBCs) contribute to hemostasis through active and passive mechanisms including increased blood viscosity, mechanical margination of platelets against the vessel wall (13), ADP-dependent enhancement of platelet aggregation (14), improved fibrin network organization (15), and nitric oxide scavenging that increases vascular tone and disinhibits platelets (16). All of these mechanisms suggest that higher hematocrits should contribute to hemostasis and ideally be represented by VETs. However, previously published *in vitro* studies have demonstrated that the clot maximum amplitude measured on a mechanical TEG (TEG 5000) has a paradoxical inverse relationship with the hematocrit (17). Small studies in clinical patients have also found increased apparent fibrinogen function in VETs in patients with anemia (18, 19). The study hypothesis was that this paradoxical relationship would be preserved in a large clinical sample, and that both mechanical and acoustic (TEG 6s) platforms would demonstrate the same confounding.

## 2 Materials and methods

This study screened all clinical TEGs from a single academic hospital for samples in which hematocrit was drawn at the same time. Specifically, samples were included if they had any mechanical (TEG 5000^TM^; Haemonetics, Boston, MA, USA) or acoustic (TEG 6s^TM^) (19) TEG drawn within 5 min of a hematocrit or hemoglobin, and the results were available in the electronic health record. All times were based on the time of sample draw. All TEGs were performed in the core laboratory. All mechanical TEGs were activated with calcium and kaolin. Acoustic TEGs were run using either the “Trauma” (Global Hemostasis with Lysis^®^) cartridge or the Global Hemostasis (with Heparinase)^®^ cartridges. In either case, we recorded the MA from the “rapid” TEG channel, which is activated with calcium, kaolin, and tissue factor.

Hemoglobin was most often measured using a commercially available blood gas analyzer (ABL800 FLEX; Radiometer, Copenhagen). Approximately 36% of all hemoglobin measurements had an accompanying hematocrit measured on a cell counter in the core laboratory; of those paired measurements, the mean hematocrit: hemoglobin ratio was 3 ± 0.1. If hemoglobin was drawn without a hematocrit, it was converted using the following formula: *hematocrit (%)* = *hemoglobin (g/dL)* × *3*. Platelet counts (Sysmex XN10s; Sysmex America, Kobe, Japan), activated partial thromboplastin time (aPTT; ACL-700 Series, Werfen, Bedford, MA, USA), international normalized ratio (INR) (19), and Clauss fibrinogen level (19) drawn within 5 min of the index TEG were also recorded, as were age and sex assigned at birth. All the study procedures were approved by the Institutional Review Board of the University of Wisconsin (approval number 2022-1330).

The primary analysis consisted of two multivariate linear models of maximum amplitude: one for the mechanical TEGs and one for the acoustic TEGs. A mixed-model structure was used to account for more than one sample drawn from the same patient. In both models, the platelet count and Clauss fibrinogen levels were included *a priori* because they were expected to be the primary predictors of the maximum amplitude. All variables were reviewed for normality; if a significant skew was present, the variables were log-transformed and centered prior to inclusion in the model. Other significant variables in the univariate analyses were also included in the preliminary models. These variables were added using both forward and backward selections, and any discrepancies in the final model composition were resolved using the Akaike Information Criterion. Secondary analyses separately modeled the effect of hematocrit on TEG R-time, K-time, and alpha angle for both the mechanical and acoustic platforms. TEG R-time was available for all TEG samples; K-time and alpha angle were available for all mechanical samples and those acoustic samples run using the Global Hemostasis (with heparinase) cartridge.

To verify the results of the multivariate model, a subgroup analysis was planned for patients with normal values on conventional coagulation tests. The intent of this subgroup analysis was to ensure that any relationship between the maximum amplitude and hematocrit observed in the primary analysis was not the result of confounding factors due to the association of anemia with coagulopathy in the setting of bleeding. A simple linear model of the maximum amplitude as a function of the hematocrit was planned without any adjustment for conventional coagulation test values. Normal ranges for conventional coagulation tests were defined as a platelet count ≥150 × 10^3^ cells/μL, an INR ≤ 1.3, and a Clauss fibrinogen ≥200 mg/dL. It was anticipated that at least a fraction of the study samples would meet these criteria, as it is routine practice at the study hospital to draw simultaneous TEGs and conventional coagulation tests at the beginning of all major cardiac, vascular, and liver surgeries to establish a baseline.

All results were analyzed and reported using conventional statistical methods. Continuous variables were reported as means with standard deviations and categorical results as counts with percentages. Univariate comparisons between continuous variables were made with Student's *t*-test or simple linear regression, and between categorical variables with Pearson's χ^2^ test. Statistical significance was set at *p* < 0.05. Any variables significant at *p* < 0.1 level were considered for inclusion in multivariate models.

## 3 Results

There were 2,507,486 available health records during the study period. After applying the inclusion and exclusion criteria, there were 7,176 samples with complete TEG and conventional coagulation test data (6,384 mechanical, 744 acoustic, 48 both) from a total of 2,849 unique patients between November 2006 and July 2023. Among these, there were 793 samples (660 mechanical, 130 acoustic, and 6 both), and the accompanying platelet count, INR, and Clauss fibrinogen were all within their normal ranges. The sample and patient characteristics are summarized in Table 1.

**Table 1:** Sample characteristics for thromboelastograms with simultaneous hematocrit (*n* = 7,176).

	**Mechanical**	**Acoustic**
	***n** =* 6,432^*^	***n** =* 792^*^
**Thromboelastogram values**		
MA (mm)	52 ± 13^†^	55 ± 8
R (min)	7 ± 4	8 ± 1
Alpha angle (deg)	68 ± 9	63 ± 11
K time (min)	3 ± 2	2 ± 1
Hematocrit (%)	28 ± 6	29 ± 6
**Coagulation test values**		
International normalized ratio (INR)	1.9 ± 1.0	1.6 ± 0.6
aPTT (sec)	50 ± 24	39 ± 16
Clauss fibrinogen (mg/dL)	210 ± 120	264 ± 130
Platelet count (10^3^/mm^3^)	106 ± 74	124 ± 72
All coagulation values within normal range^‡^ (*n*, [%])	666 [10]	136 [17]
**Patient Characteristics**		
Female sex (*n*, [%])	2,085 [32]	252 [32]
Age at time of sample (years)	56 ± 14	58 ± 15
Samples per patient (median [IQR])	2 [1, 2]	1 [1, 2]

^*^Includes 48 samples for which both mechanical and acoustic tests were performed. Mechanical tests were performed with the calcium kaolin (CK) reagent, and acoustic tests were performed with the calcium-kaolin-tissue factor (CRT) reagent.

^†^Mean ± standard deviation unless otherwise noted.

^‡^Defined as platelet count ≥150 × 10^3^ cells/μL, an INR ≤ 1.3, and a Clauss fibrinogen ≥200 mg/dL.

In unadjusted analyses, hematocrit was positively associated with maximum amplitude on both the mechanical (β = 0.35 mm/%, 95% confidence interval [CI] 0.30, 0.40; *p* > 0.001) and acoustic platforms (β = 0.29 mm/%, 95% CI 0.20, 0.38; *p* > 0.001). Platelet count, Clauss fibrinogen, INR, and aPTT were all significantly associated with the maximum amplitude on both platforms, and all tests were significantly associated with hematocrit. Among the patient characteristics, age was significantly associated with the maximum amplitude on both platforms, and sex was significantly associated with the maximum amplitude on the acoustic platform but not on the mechanical platform.

The two primary analytical models are listed in Table 2. In the primary analysis of TEG samples run on the mechanical platform, hematocrit was negatively associated with the maximum amplitude after adjusting for platelet count, Clauss fibrinogen, and aPTT, and accounting for repeated measures from the same patient. In the primary analysis of samples run on the acoustic platform, hematocrit was also negatively associated with the maximum amplitude after adjusting for platelet count, Clauss fibrinogen, and age, and accounting for repeated measures from the same patient. Patient sex and INR were not significantly associated with the maximum amplitude in either model.

**Table 2:** Primary analyses using mixed multivariable models of mechanical thromboelastogram (kaolin) and acoustic thromboelastogram (kaolin & tissue factor) maximum amplitude as a function of hematocrit and other factors among all eligible samples.

	**Parameter estimate (95% confidence interval)**	***p***
**Model 1: mechanical thromboelastogram**		
**(*****n** =* **6,432)**		
**Fixed effects**		
Hematocrit	−0.23 mm/% (−0.25, −0.20)	<0.001
Platelet count	0.07 mm/10^3^/mm^3^ (0.06, 0.07)	<0.001
Clauss fibrinogen	0.04 mm/g/dL (0.04, 0.05)	<0.001
aPTT	−0.05 mm/sec (−0.07, −0.05)	<0.001
**Repeated effects**		
Patient (intraclass correlation)	0.33 ± 0.02	
**Model 2: acoustic thromboelastogram**		
**(*****n** =* **792)**		
**Fixed effects**		
Hematocrit	−0.26 mm/% (−0.31, −0.21)	<0.001
Platelet count	0.07 mm/10^3^/mm^3^ (0.06, 0.07)	<0.001
Clauss fibrinogen	0.03 mm/g/dL (0.03, 0.03)	<0.001
Age	0.03 mm/year (0.01, 0.05)	0.010
**Repeated effects**		
Patient (intraclass correlation)	0.44 ± 0.04	

**Figure 1:**
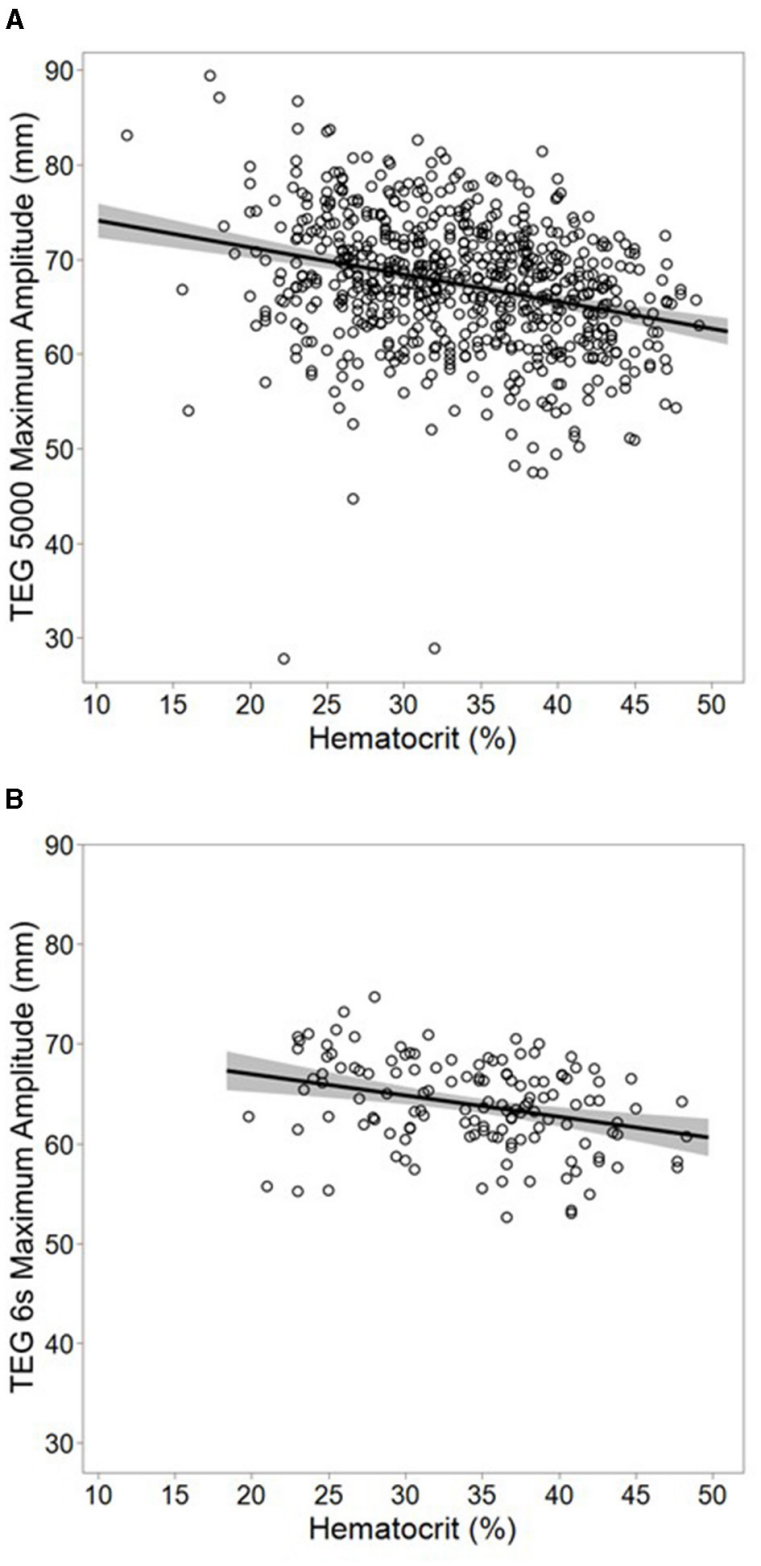
Subgroup analysis using multivariable models of mechanical [**(A)**; *n* = 666] and acoustic [**(B)**; *n* = 137] thromboelastogram maximum amplitude among samples with only normal conventional coagulation values. Plots represent actual values of maximum amplitude vs. hematocrit, overlaid with a simple linear regression model of the two variables. The band around the line represents the 95% confidence interval for the model. Hematocrit was inversely associated with maximum amplitude after adjusting for patient age on both mechanical (β = −0.29 mm/%, 95% CI −0.21, −0.36; r^2^ = 0.08, *p* < 0.001) and acoustic (β = −0.22 mm/%, 95% CI −0.10, −0.33; r^2^ = 0.09 *p* < 0.001) platforms. No adjustment was made for any other factors. Normal ranges for conventional coagulation tests were defined as a platelet count ≥150 × 10^3^ cells/μL, an international normalized ratio ≤ 1.3, and a Clauss fibrinogen ≥200 mg/dL.

In the subgroup analysis of patients with only normal conventional test values, hematocrit was negatively associated with the maximum amplitude as measured on both the mechanical (β = −0.29 mm/%, 95% CI −0.21, −0.36; *p* < 0.001, Figure 1A) and acoustic platforms (β = −0.22 mm/%, 95% CI −0.10, −0.33; *p* < 0.001, Figure 1B). No adjustments were made for conventional coagulation test values in this subgroup.

In secondary analyses, hematocrit was associated with other fibrinogen-associated TEG parameters. On the mechanical platform, there was an inverse association with the alpha angle (β = −0.17 mm/°, 95% CI −0.20, −0.14; *p* < 0.001) and a positive association with the K time (β = 0.04 mm/min, 95% CI 0.03, 0.05; *p* < 0.001) after controlling for Clauss fibrinogen levels and multiple samples from the same patient. No association with R time was observed after controlling for aPTT. The effects were of similar direction and magnitude for the acoustic platform; however, none were statistically significant (data not shown).

Among acoustic samples, we noted that 619 (78%) were run using the Global Hemostasis with Lysis^®^ cartridge, 105 (13%) were run using the Global Hemostasis (with Heparinase)^®^ cartridge, and 68 (9%) were run using both cartridges. When the multivariable model of acoustic MA was stratified by cartridge type, we found no meaningful difference in the magnitude or the direction of the relationship with hematocrit compared to the primary, pooled analysis (Lysis β = −0.21 mm/°, 95% CI −0.26, −0.16; Heparinase β = −0.26 mm/°, 95% CI −0.42, −0.11).

## 4 Discussion

In a large retrospective clinical sample, the TEG maximum amplitude had an inverse relationship with hematocrit after controlling for fibrinogen and platelet concentrations. In general, there was a reduction in maximum amplitude of approximately 0.25 mm for each percentage point increase in hematocrit, and this was consistent across a wide range of hematocrits and coagulation test values and on both mechanical and acoustic testing platforms. Although the magnitude of the effect is not large, it is still paradoxical because higher hematocrit values are associated with less bleeding and increased coagulation potential, which would ideally be reflected in a higher clot shear modulus, as measured by TEG.

Normal clots are formed by the interplay of clotting enzymes and cofactors, platelets, RBCs, the physics of shear flow, blood pressure, vascular tone, temperature, pH, and the extent of endothelial injury. RBCs promote and support hemostasis *in vivo* (10–16), and some of the prothrombotic effects of RBCs will still function in a TEG chamber. However, because the testing volume of whole blood in TEG is fixed, any increase in the hematocrit must be accompanied by a decrease in the total mass of available fibrinogen. Fibrinogen loss may also be the result of differences in handling between whole blood TEG samples and conventional coags tests, which are performed in plasma. Fibrinogen is a more potent contributor to the clot shear modulus than platelet quantity or RBCs (20); thus, a decrease in the total fibrinogen mass would result in a decreased maximum amplitude. In this situation, the increase in the hematocrit and decrease in total fibrinogen mass would also result in a lower alpha angle and increased K time, which was observed in this study (1). Although the current study can only identify statistical associations, displacement of fibrinogen mass appears to be the most likely hypothesis to explain how hematocrit would be negatively associated with maximum amplitude, regardless of the true hemostatic potential of the whole blood.

## 5 Conclusions

A study of 7,176 clinical TEG and hematocrit samples suggests that TEG misrepresents the contribution of RBCs to normal hemostasis. Given the dominant contributions of fibrinogen and platelets to clot shear modulus, this effect is likely to be minor in most patients with bleeding. Further research is required to quantify the clinical implications of this phenomenon.

## Data Availability

The raw data supporting the conclusions of this article will be made available by the authors, without undue reservation.
